# Impacted Bones: Can Extant Primates Help Identify Tool Use in Early Hominins?

**DOI:** 10.1002/evan.70042

**Published:** 2026-07-19

**Authors:** Rachel M. Hurwitz, Katarina Almeida‐Warren, Susana Carvalho, Thomas A. Püschel

**Affiliations:** ^1^ School of Anthropology and Museum Ethnography, Institute of Human Sciences University of Oxford Oxford UK; ^2^ Interdisciplinary Center for Archaeology and Evolution of Human Behaviour (ICArEHB) FCHS University of Algarve, Campus de Gambelas Faro Portugal; ^3^ CIBIO‐BIOPOLIS, Centro de Investigação em Biodiversidade e Recursos Genéticos Campus de Vairão Porto Portugal; ^4^ Department of Science Gorongosa National Park Sofala Province Mozambique

**Keywords:** biological asymmetry, bone morphology, capuchins, chimpanzees, laterality, lithic tool use, macaques, nut‐cracking, percussive tool use, primate tool use

## Abstract

Despite recent advances pushing back the earliest record of tool use, how and when it first emerged in hominins, and the extent to which it featured amongst the numerous co‐existing species, remain critical questions in paleoanthropology. In addition to analyzing fossils and lithic toolkits, novel strides in understanding this might be made by studying the skeletons of the great apes, as well as more distantly related tool‐using primates and applying resultant findings to the fossil record via predictive modelling. We review current methods of extrapolating tool‐use capabilities in extinct and extant taxa and provide an innovative methodological framework to analyze osteological remains for evidence of percussive tool use, using chimpanzees as a case study. We propose a shift in current methodological practice, wherein both sides of the body, rather than just one, should be routinely scanned to understand of the effect percussive tool use on primate bone. Key species, including long‐tailed macaques (*Macaca fascicularis* ssp.), capuchins (*Sapajus* and *Cebus* sp.) and chimpanzees (*Pan troglodytes* ssp.), all of whom engage in lithic percussion, should be the work's focus. Due to bone's dynamic ability to respond to mechanical strain, percussive forms of tool use, like nut‐cracking, could cause morphological changes in the dominant hands of tool users as compared to their non‐dominant hand, or the hands of primates who do not engage in percussive behaviors. However, this potential “damage signature” has not been quantified. Thus, we propose a methodology for examining the effect of percussive tool use on primate bone by scanning skeletal remains bilaterally, analyzing directional asymmetry, and using machine learning to discern patterns of variation. Long‐term, our proposed methodology may be extrapolated to examine the lasting impact of tool use on the bones of extinct hominins.

## 1 Introduction

Modern humans (*Homo sapiens sapiens*) are considered unique in numerous ways, including a propensity for tool use, highly dexterous hands, strong degree of handedness, complex social structures, reliance on prosociality and social learning, high degree of encephalization, use of language, ability for cumulative cultural evolution, and bipedal locomotion, among other evolutionary singularities [1]. While many of these anatomical and cognitive characteristics are present to varying degrees throughout the primate clade, humans seem to be at the extremes of these traits, and particularly in our tendency for strong (right) handedness [2].

Human tool use is not unique in itself, as numerous other species utilize both lithic (stone) and organic implements to accomplish various tasks [3]. While less than one percent of Earth's species are known to engage with tools, this still accounts for many species in distantly relates clades, including corvids, parrots, various primates, aquatic mammals, elephants, and otters [3]. Plentiful primate species in various families (Cercopithecidae, Cebidae, Hominidae) including capuchins (*Sapajus* sp. and *Cebus* sp.), macaques (*Macaca* sp.*)*, chimpanzees (*Pan troglodytes* ssp.), and orangutans (*Pongo* sp.) all use tools regularly, with anecdotal evidence of other species such as baboons (*Papio ursinus*), gorillas (*Gorilla* sp.), and bonobos (*Pan paniscus*) using tools, almost exclusively in captivity and most frequently to obtain food rewards or during experiments [3, 4]. However, *H. sapiens*' consistent construction of, reliance on, and functional requirement on tools for survival, is distinctive [1, 4].

When hominins began routinely making and using tools is a frequently debated topic in palaeoanthropology, with recent evidence implying that tool use began long before the emergence of the genus *Homo* over 3 million years ago [5, 6, 7]. Debate surrounding this topic stems from the fact that lithic tools, although often found within similar geographic and temporal contexts, are rarely discovered contemporaneously with in situ preserved hominin fossils. As a result, determining which hominin species manufactured and used these tools remains largely inferential [8]. Therefore, numerous studies have examined whether the morphological features of fossilized hominin hands would have allowed the degree of manual dexterity associated with the type of lithic tool use seen in *H. sapiens* [9]. Additionally, organic (plant‐based) tools, which likely comprised a significant portion of ancient toolkits, are not found in the archaeological record until 430 Ka [10], meaning there is even less concrete evidence for when or how hominins began employing organic implements to aid in accomplishing tasks [11]. While it is often stated that behaviors themselves cannot fossilize, physical traces may persist, a principle central to ichnology, the study of behavioral evidence preserved in the form of trace fossils. Such traces may include potential indicators of tool use, like cut marks on bone or teeth, scars on living plants from raw material extraction, fractured shells of cracked nuts, or the ashy remnants of a fire pit [12, 13].

**Figure 1:**
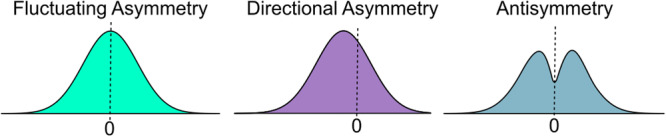
Main forms of biological asymmetry in bilaterally symmetric organisms. Fluctuating asymmetry reflects levels of developmental stability and refers to minute, random deviations from the expected sameness in bilateral traits due to environmental, cellular, or genetic stressors. Directional symmetry is the systematic and regular variance between two halves of an organism in a predictable fashion within a population. Antisymmetry occurs between individuals of a population, with some displaying a significant left bias in a trait and others displaying a significant right bias, leading to a bimodal distribution.

Modern humans' strong degree of cross‐cultural rightward manual lateralization, termed handedness [14], something thought to be connected to our propensity for tool use [15], has been linked to the development of skeletal asymmetries, particularly in cortical bone of the upper limbs, as early as 9 years of age [16]. Still, there is some debate regarding the extent and cause of upper limb directional asymmetries found [17]. Directional asymmetry, as described in Figure 1, refers to is the systematic and regular variance between two halves of a bilaterally symmetric organism, wherein one side develops larger or differently than the other in a predictable fashion [18]. These asymmetries can be large, such as the additional lobe in the right lung compared to the left, or more subtle variations in the shape and size of structures [18]. Although directional asymmetry is not solely a lasting physical result of manual lateralization in the upper limbs, it is often studied as a proxy for lateralization in humans—yet this methodology has seldom been applied to non‐human species [19, 20, 21, 22].

Because bone tissue is mechanosensitive and tends to remodel in response to the various strains it endures throughout the course of an individual's life, our highly lateralized manual behaviors can influence bone morphology [23, 24]. Numerous observational and experimental studies both in humans and other mammals have indicated the strong effect of activity on various aspects of osseous morphology, which will be elaborated on in Sections 6 and 7 [25, 26, 27, 28]. Importantly, aside from teeth, bone is the most frequently fossilized tissue, meaning the effects of human handedness may be visible in the fossil record. Thus, studying the upper limb, and particularly the hand and its skeletal scaffolding, is a critical area of primatology and paleoanthropology, as it may provide insight not only into the various evolutionary pressures that shaped the bones of primate hands in diverse directions, but also the ways bone structure has been affected by habitual loads and activity patterns unique to that species or individual, including potential manual specializations. Therefore, determining the level of directional asymmetry present in the bones of extant hominids may help us make inferences as to when and why hominins began to utilize tools, particularly percussive tools, and when our incredibly strong degree of manual lateralization evolved [29]. Here, we review how tool‐use capabilities are typically studied in extinct taxa and provide an innovative methodological framework to collect and analyze extant and extinct osteological remains for potential tool‐use traces. Using chimpanzees as a case study, we aim to help clarify the relevance and applicability of this suggested methodology wherein bones from both hands of the same individual primates are compared for osteological changes that may reflect percussive tool‐use behaviors.

## 2 Defining Non‐Human Tool Use

While tool use is relatively rare in the animal kingdom, it been observed to some extent in numerous non‐human primate species, including in all extant great apes (Hominidae), capuchins (*Cebus* sp. and *Sapajus* sp.), macaques (*Macaca* sp.) and baboons (*Papio* sp.), as well as non‐primates including aquatic mammals like bottle‐nose dolphins and otters, and birds like corvids and parrots [3]. For many years, there has been widespread discussion regarding the definition for, and categories of, what constitutes tool use in non‐human species, some of which can be found in Table 1. These have varied in terms of the importance of whether the potential ‘tool’ is dynamic or static, the necessity of and extent to which the tool is manufactured, how the tool extends the abilities and the efficiency of the animal in the given task. For further discussion of the complexities of defining tool use in non‐human animals, please see [30, 31, 32, 33, 34]. Importantly, the interpretation of a primate's cognitive and manual abilities, as well as the origin of tool use itself, may vary based upon which definition is adhered to.

**Table 1:** A selection of some of the most frequently used definitions of tool‐use in non‐human animals or primates.

Definition	Reference
The use of an external object as a functional extension of mouth or beak, hand or claw, in the attainment of an immediate goal.	Van Lawick‐ Goodall [35]
Tool use is the external employment of an unattached environmental object to alter more efficiently the form, position, or condition of another object, another organism, or the user itself when the user holds or carries the tool during or just prior to use and is responsible for the proper and effective orientation of the tool.	Beck [36]
Tool use is the exertion of control over a freely manipulable external object (the tool) with the goal of (1) altering the physical properties of another object, substance, surface or medium (the target, which may be the tool user or another organism) via a dynamic mechanical interaction, or (2) mediating the flow of information between the tool user and the environment or other organisms in the environment.	St. Amant & Horton [31]
The external employment of an unattached or *manipulable attached* environmental object to alter more efficiently the form, position, or condition of another object, another organism, or the user itself, when the user holds and *directly manipulates* the tool during or prior to use and is responsible for the proper and effective orientation of the tool.	Shumaker et al. [3]
The use of an external object by a nonhuman primate to extend the function of its hand, foot, or mouth to help attain a desired outcome.	Silva & Silva [37]

Most succinctly, for the purposes of this article, tool use entails the dynamic manipulation of an object which is not part of the animal's body in order to help it achieve a particular goal [3]. The tool may or may not be modified before use, but it typically adds some functionality to the animal's body and aids in completing the task in some way [3]. For example, if a chimpanzee has a goal of eating termites hidden in a mound, they could hold a stick in their hand, insert that stick into the termite nest, wait for the termites to bite the stick, extract the stick with the insects, and eat said insects. By using the stick, the chimpanzee can access the nutritional resource (in the form of termites) when its finger likely would not fit into the nest holes, and it is able to do so with less risk of being bitten by defensive termites. This constitutes extractive foraging, wherein an animal removes a nutritional resource that is “hidden” in some way, such as encased within a shell or concealed underground [38]. In primates, tool use can be grouped into broad contextual categories, which tend to be related to feeding, hygiene, communication or social interactions, and protection [39]. The utilization of sticks in the aforementioned termite example, and the use of stones to crack open nuts, would fall under the tool‐assisted foraging category [37]. Examples of tool use in the other categories include using moss or leaves for fluid transportation (feeding), using an object to groom (which can fall into either the social or hygienic category), manipulating large leaves to act as umbrellas (for protection from the elements), throwing stones as weapons (combative social interactions), and assistance in maneuvering or climbing [3, 39]. There is debate regarding whether behaviors such as nest‐building or communicative gestures using non‐body objects should be included as forms of tool use, which is based on the “objective” of the task and the observer's interpretation of how and why the item is being utilized, however an in depth discussion of these distinctions goes beyond the scope of this review [3].

**Figure 2:**
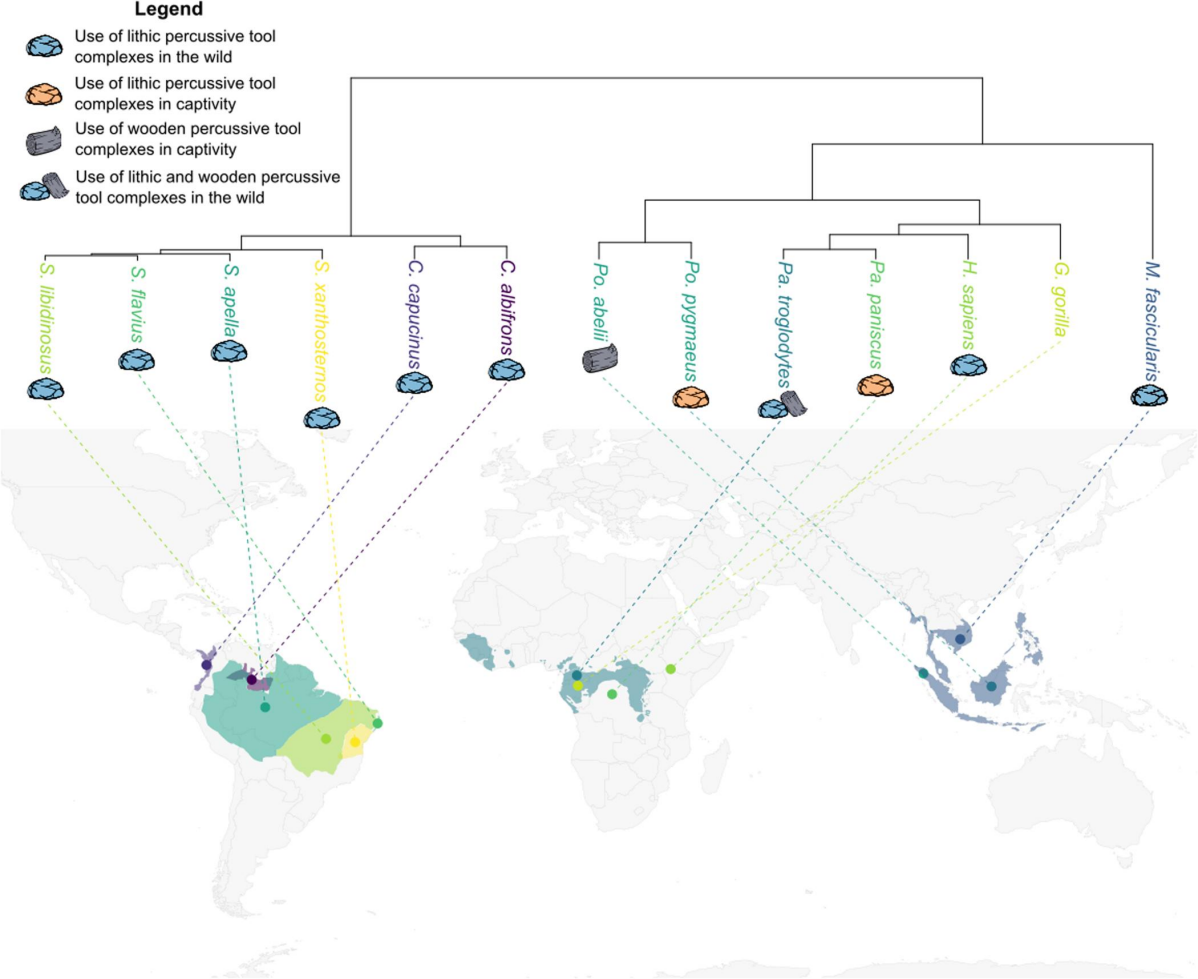
Phylogenetic Distribution of percussive tool use among anthropoid genera. This includes lithic on lithic, or lithic on wood tool complexes. Use of stone as projectiles are not included in this phylogeny, nor are cracking behaviors wherein only an anvil is used (i.e. hitting a coconut on a rock to fracture its shell). Range data from IUCN Red List 2024. The IUCN Red List of Threatened Species. Version 3.1. https://www.iucnredlist.org. Downloaded on 05‐03‐2026 [47]. The coordinates for *Homo sapiens* reference the earliest site of the most widely accepted human remains from the Omo‐Kibish Formation [48]. References for lithic percussive use for each genera are as follows: ***Pongo*,** Bandini et al. [46]; Motes‐Rodrigo et al. [49]; *Pan paniscus*, Neufuss et al. [41]; *Pan troglodytes*, Luncz et al. [50]; *Macaca fascicularis,* Gumert et al. [44]; *Cebus capucinus*, Barrett et al. [38]; *Cebus albifrons*, Araujo P et al. [51]; *Sapajus apella*, Struhsaker TT, Leland L. 1977 [52] *Sapajus flavius*, Lima et al. [53]; *Sapajus libidinosus*, Visalberghi et al. [54]; *Sapajus xanthosternos*, Mainette et al. [55].

In order to reconstruct the evolution of hominin tool use, combined with analyzing skeletal morphology, observation of modern primate toolkits and the ways they manipulate, grasp, and engage with lithic or organic tools can serve as proxies into the potential abilities of extinct hominins [40, 41]. For this reason, studies of extant primates from different clades can complement evidence for tool use from the paleoanthropological record. Due to their close evolutionary proximity to both modern humans and extinct hominins, the great apes, and particularly the genus *Pan*, often serve as the main models for inferring the behavioral capabilities of extinct hominins [8, 42]. However, other, more distantly related primates can also serve as varied models for early forms of hominin tool use [4], providing new ways to interpret paleoanthropological remains. This is the case for capuchins, who accidentally create flakes which broadly resemble Oldowan technology [43]; macaques, who use lithic tools in coastal environments and produce flakes which also do not significantly differ from Oldowan assemblages [44, 45]; and orangutans who use numerous tools in arboreal contexts [46]. Importantly, in addition to demonstrating the range of primate species who can leave behind archeological assemblages, this also calls into question the assumption that early hominin lithics were necessarily intentionally manufactured [45]. The primate genera who have been documented engaging in forms of percussive tool use, both in the wild and in captivity, are summarized in Figure 2. While all of these primates are evolved to, and specialized for, their own ecological niches which are inherently different from those of the first hominin tool users, they may still provide insight into the potential behavioral mechanics of tool use by extinct hominins [7, 43].

When discussing lithic tools, it should be noted that while captive orangutans and bonobos have been documented utilizing cutting tools like those found in the paleoanthropological record in captivity, no extant primate species have been recorded creating then using sharp‐edged tools in the wild [49, 56]. Similarly, these species typically employ a different type of precision grip (pad to side, v. pad to pad) than modern humans when engaging with these tasks due to morphological and neurological differences, particularly through ontogeny [56, 57]. Electroencephalographic studies of lithic percussion and cutting behaviors in modern humans have demonstrated fundamental differences in the cognitive demands of the two tasks, indicating potential neurological rationale for the distinction between human lithic tool behaviors compared to modern wild primates [58]. Thus, while extinct hominids since the *Pan‐Homo* divergence would likely have been physically able of engaging in some degree precision grip‐based tool‐use [56], whether they were cognitively capable of intentional flaking followed by cutting‐based tool‐use is questionable [58].

## 3 Current Methodologies for Examining Tool Use Skills in Extinct Hominins

As a result of morphological studies, many scholars agree that various australopiths, such as *A. afarensis*, *A. sediba*, and *A. africanus*, as well as members of the *Paranthropus* clade were likely able to make and use tools in some capacity [6, 59, 60]. Yet, studies often provide contradictory results regarding the frequency and ease with which these tools could have been produced and used. Some scholars argue that morphological evidence suggests some australopiths could create and use knapped lithics in a similar manner modern humans [6, 61, 62], while others use similar analyses and evidence to suggest the exact opposite [63, 64]. For example, a recent analysis of newly discovered fossils of *Paranthropus boisei*'s hand has revealed morphologic convergence with gorillas, while featuring a more human‐like pollex (thumb) to finger ratio, which is unlike modern gorillas [59]. The authors conclude that *P. boisei* would have moved arboreally in a manner similar to gorillas, been capable of strong grip strength for the processing rough foods, and further suggest it would have been able to engage in tool use similar to that of modern humans [59]. Another recent study comparing entheseal morphology in australopiths, humans, neanderthals, and apes suggested that *A. sediba* likely used its hands in a fashion similar to members of the genus *Homo*, whereas *A. africanus* and *A. afarensis* likely routinely used its hands in ways that resembled both ape and human manipulation [60].

When examining morphological features of extinct hominins, studies oftentimes examine the external features of bone, including apomorphic (evolutionarily ‘new’ or derived) features like broad apical tufts (the flared distal end of a distal phalangeal bone in primates) which aids in dexterous gripping, or the level of curvature of phalanges, as increased curvature is beneficial for maneuvering in arboreal contexts [5]. Alternatively, they may compare aspects of morphology which are reactive to activity patterns, such as relating entheseal shape (muscle attachment sites) to the known effects of various forms of manual labor [60, 65], estimating loading patterns based on the directionality of trabecular struts [66] or modelling the distribution of stress that would be expected during lithic tool‐use in extant non‐human primates and extinct hominins [67]. For example, Kunze et al. [6] used three entheses on the 1st metacarpal (MC1) to reconstruct muscle recruitment patterns in modern humans, extant hominids, and some extinct hominins. They found that the resultant muscle recruitment patterns differed between modern humans and extant hominids, but was similar to modern humans in all extinct hominins (except *A. africanus*) studied [6]. Conversely, studies may examine evolved features such as phalangeal curvature, or the length of the pollex relative to the other digits, as proxies for how much the hand was used in (likely arboreal) locomotion or how human‐like it was, and thus whether it could complete delicate, precise manipulation, or they use biomechanical methods to model potential dexterity [5, 6, 68], rather than analyzing the more‐frequently internal features that actively remodel as a result of loading patterns, such as trabecular morphology or cross‐sectional geometry. It is rare for fossils from both sides of body of the same individual to be found [5], making bimanual studies understandably scarce, however, even when looking at extant primates, research seldom compares bones from both sides of the same individual.

Importantly though, asymmetric variation has been observed using various methods in the few studies which have investigated this in both humans and apes, as well as in experimental studies of other mammals [69]. Numerous studies have demonstrated that physical actions associated with lateralized sports like cricket, baseball, and tennis can affect macro‐ and micro‐architectural metrics of long bones [25, 28, 70]. In a more generalist population, a study of human humeri from various geographic origins found right‐sided asymmetry in terms of length, weight, and mid‐shaft circumference, but more variable patterns of asymmetry in terms of the angle of torsion [71]. Another study examining human humeri and second metacarpals (MC2), found entheses were more likely to be asymmetric on both bones, although they did not report a trend in directionality of the asymmetries found [72]. However, another study comparing the enthesis for the opponens pollicis muscle on the MC1 in various primates including macaques, gibbons, gorillas and humans found that rightward directional biases were present only in humans, which the authors suggest is a result of the lower degree of lateral hand‐use bias found in non‐human primates [73]. Studies examining total subperiosteal area in the midshaft of human and chimpanzee humeri and MC2 found that all human populations studied demonstrated a right‐bias in both bones, whereas chimpanzees tended toward left‐lateralization in the humerus but right‐lateralization in the MC2 [19, 74]. Directional asymmetries have also been identified in an ontogenetic series of captive old world monkey (*Macaca mulatta*) [21], and in the basic geometry of the humerus and ulna in adults of the same species [75], as well as in adult new world monkey, the cotton‐top tamarin (*Saguinus oedipus*) [76], albeit only in the lower limb in the latter.

Unilateral studies examining the internal, trabecular structure of bones, which is particularly reactive to mechanical strain and therefore may be uniquely suited to helping infer behavioral capabilities of fossil hominins, are rare as well, likely due to the logistical challenges and cost of acquiring microCT scans, though they do exist [9, 23, 62, 77]. These studies are constrained by the fact that taphonomic distortion can affect various trabecular measurements (i.e. bone volume fraction, trabecular thickness, trabecular spacing, etc.) meaning that observed differences could be reflective of preservation rather than actual biological differences resultant from differential behaviors. Similarly few studies have looked at potential asymmetries present in internal bone microarchitecture in humans bilaterally, and only one small study has done so in any of the extant great apes, which found the MC1 reflected rightward directional asymmetries in humans but leftward ones in chimpanzees [20, 68]. While the rate of osseous remodeling varies as a result of hormonal and life history factors, trabecular bone tends to remodel at a much faster rate than cortical, meaning it can be more sensitive and responsive to behavioral loads and may be particularly relevant to this sort of work [78, 79]. Alternatively, however, it has been suggested trabecular analyses may be less adept at capturing directional asymmetries as a result of lateralization in the carpals and metacarpals in modern humans [80]. Limited work has used Finite Element Analysis (FEA)—a virtual computational technique that simulates how complex structures withstand physical loads by breaking them down into smaller, simpler “elements”—to model the potential stress distribution patterns of hammerstone use on the proximal pollical phalanx in humans, neanderthals, and extant apes unilaterally [67]. However, as with many model‐based methods, FEA studies are often constrained by the assumptions and simplifications required during model development. Validating these models against experimental data can help mitigate the impact of these limitations [65].

While several studies have uncovered specific microwear signatures when investigating use‐wear from percussion on both organic anvils as well as lithic hammers and anvils themselves used by both humans and primates [42, 81, 82, 83], the effects on the tool user themselves are not examined. The tools discussed are often limited to human‐knapped, manufactured tools, like those used for hunting or butchering carcasses. Organic tools, or unmodified lithics, such as those used to crack open nuts by modern primates and in the archaeological past [82], are less frequently analyzed, even though the latter of which can unintentionally create flakes that are morphologically similar to early purposefully knapped technologies [43]. It is recognized that the variation in individual skill is reflected in primate tool use, such as how the technique and skill of capuchins affects unintentional flaking of stones when nut‐cracking [81, 83], and that chimpanzees in the same community consistency crack at differing efficiencies [84, 85]. Ultimately, this lack of study into directional asymmetries leaves a gap in our understanding of the effect of tool use on primate bone, particularly those who engage in lithic percussive tool use.

## 4 Percussive Tool Use in Primates

**Figure 3:**
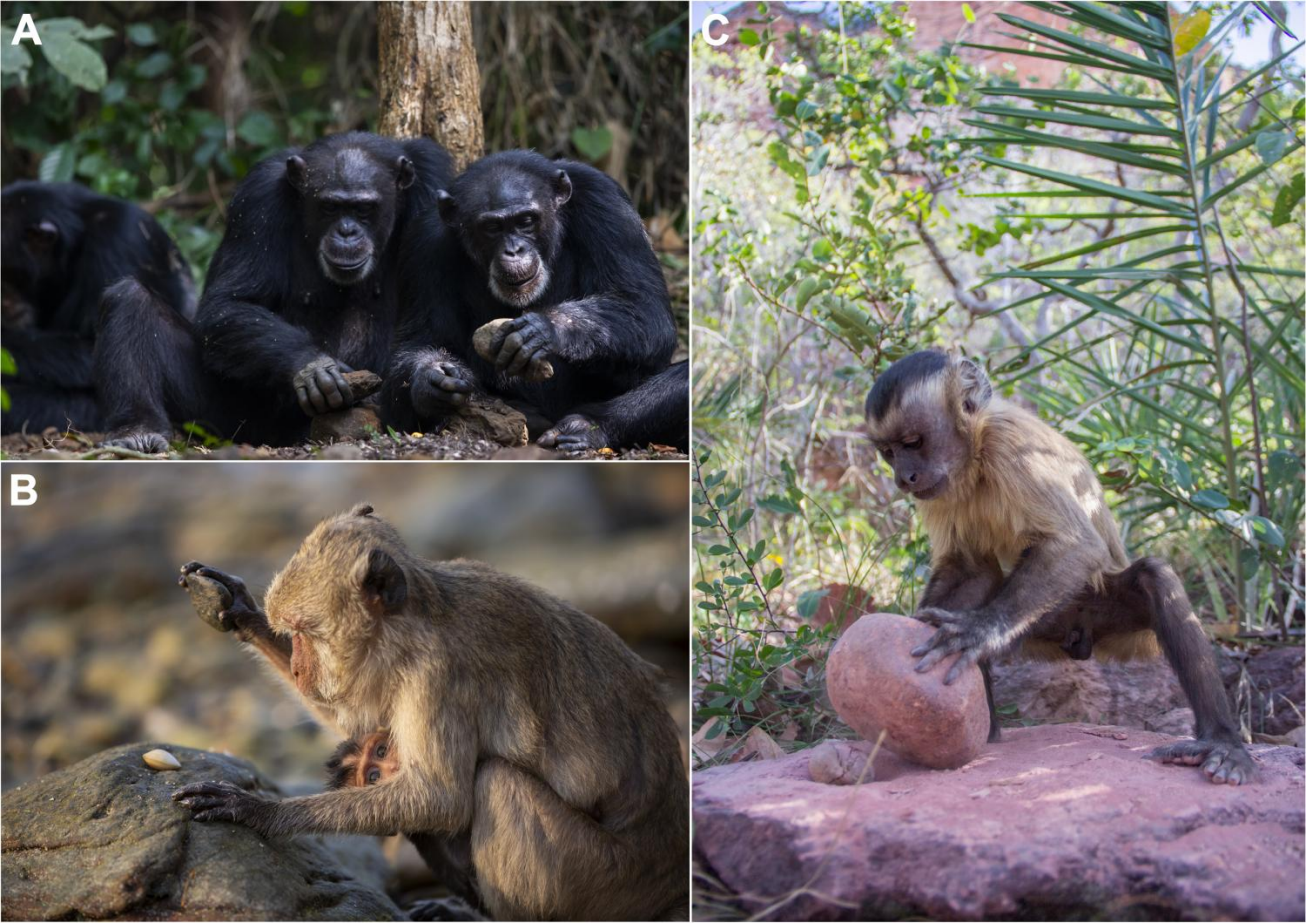
Primates engaging in lithic tool use. A: A group of Bossou Chimpanzees (*Pan troglodytes* verus) using a lithic hammer and anvil to crack nuts in the outdoor laboratory, Photo Credit: Cyril Ruoso. B: A female long‐tailed macaque (*Macaca fascicularis*) using a lithic hammer and anvil to break open a mollusc, Photo Credit: Cyril Ruoso. C: A male bearded capuchin (*Sapajus libidinosus*) cracking a piassava palm nut at Fazenda Boa Vista, Photo Credit: Luca Antonio Marino.

Lithic percussive tool use in non‐human primates has been defined as “pounding, using a hammer and/or a hammer‐and‐anvil technique in order to crack open foodstuffs,” [86]. This is with the known exception of Kanzi, a captive bonobo who was taught to flake stone tools similar to Oldowan instruments [87] and some experiments in which other captive bonobos and orangutans have been able to use lithic cutting tools [49, 56]. While other primate species use various plant‐based tools, the only primates with long‐term evidence of percussive lithic tool use in the wild are capuchins, macaques, and chimpanzees [4]. In all these species, stone tools, occasionally used in combination with organic ones, are employed for extractive foraging purposes, most frequently to remove the kernel from varied species of nut‐producing plants, or to acquire other nutritional resources as shown in Figure 3 [83].

### 4.1 Macaca

Of the old‐world monkeys, multiple species of macaque such as *Macaca silenus*, *M. radiata*, and *M. fuscata*, and subspecies of long‐tailed macaque (*Macaca fascicularis fascicularis*, *M. f. umbrosus*, *M. f. aurea)* have been documented engaging in extractive foraging and food preparation behaviors, such as washing sweet potatoes, rubbing off bitter pulp using tree bark, or processing coconuts [88]. However, only *M. fascicularis* ssp. have been documented engaging in lithic percussion. In intertidal habitats in Thailand, *M. f. aurea* has been documented conducting extractive foraging using stone hammers to crack open the shells of nearly 50 marine species, primarily of aquatic mollusks and oysters, as well as some crustaceans, and plants such as sea almonds (*Terminalia catappa*) [44, 89]. Regarded as a generalist species, it seems unsurprising that these macaques opportunistically exploit such a wide variety of prey species, even though a few species are processed and eaten at much higher rates (such as the rock oyster, *Saccostrea cucullata*; chameleon nerite, *Nerita chamaeleon*; drill rock shell snail, *Thais bitubercularis*; tooth‐lipped snail, *Monodonta labio*).

When engaging in lithic percussion to open shelled aquatic species, macaques may utilize one or both hands to hold the hammerstone [90]. Whether one or both hands are used varies based on the size of the hammerstone, which tend to be smaller “axe” hammers for cracking sessile oysters cemented to coastal rocks, or larger “pound” hammers for cracking sea almonds [44, 90]. In inland forest habitats of another island in Thailand, *M. fascicularis* have begun to crack non‐native oil palm nuts (*Elaeis guineensis*), the same variety which chimpanzees have been documented cracking for decades, recently introduced to the island by human farmers [91, 92]. Similarly, anvil and hammerstone re‐use is common, with macaques transporting tools and prey [93], leading to large amounts of macro‐ and microwear including flake removals, pitting, and flattening of the surface [89, 92]. Importantly, the accidental production of sharp‐edged flakes through this extractive foraging can result in objects which could easily be misinterpreted as purposeful and anthropogenic in nature, making them hard to distinguish from the human archaeological record [45].

### 4.2 *Sapajus* and *Cebus*

Although there are limited reports of other new world monkeys using organic tools, capuchin monkeys of both genera (*Sapajus* and *Cebus*) are the most well‐documented tool using new world monkey in the wild [13, 39]. Even though robust capuchins (*Sapajus* sp.) are thought to be more adapted to extractive foraging than gracile capuchins (*Cebus* sp.) due to the ecological availability of nutritional resources in their environments, species from both genera have been reported engaging in lithic tool use to some degree in the wild [94].

Bearded capuchin monkeys (*Sapajus libidinosus*, formerly *Cebus libidinosus*) are well‐studied for their percussive tool use. In savannah habitats, they use stones to crack various species of nuts and seeds, and in dry forest and woodland habitats, they use stones to break down tubers, or small vertebrate prey [95, 96]. They also engage in stone‐on‐stone percussion for aggressive or sexual displays, often licking at the stone's fracture point afterward, possibly to ingest the pulverized rock or lichens [97, 98]. Interestingly, and like with long‐tailed macaques, this behavior can create flakes which are morphologically similar to intentionally flaked hominin tools in the archeological record [97]. Golden‐bellied capuchins (*Sapajus xanthosternos*) in Northern Brazil have been recorded using lithic hammers to open the enclosed fruits of three species (*Acrocomia aculeata*, *Cnidosculus pubescens*, and *Syagrus oleracea*) during only the dry season [55]. Blond capuchins (*Sapajus flavius*) have recently been reported using hammerstones in the Caatinga dry forest, although it is unclear which plant species they processed, or which individuals in the group were engaging with the tools [53]. While the manipulative and cognitive abilities of tufted capuchins (*Sapajus apella*, formerly *Cebus apella*) with tools have long been studied in captivity, there are fewer examples of them using lithics in the wild [94, 99]. Most reports of this species engaging in nut‐cracking in the wild do not involve any lithics, as they instead hit tough fruit on tree branches, with lithic‐based nut‐cracking reported only in semi‐captive groups [100, 101]. Similarly, crested capuchin monkeys (*Sapajus robustus*) crack open nuts using stones and other hard objects, however this has only been reported in captivity, and not all of the individuals who were reported to attempt this behavior were successful [102].

Gracile capuchins have far more limited evidence of lithic percussion in the wild, although a few species have been documented using lithics for extractive foraging. Much like macaques in tidal habitats, some groups of Panamanian white‐faced capuchins (*Cebus capucinus imitator*) use lithic tools to crack open mollusks such as snails, as well as crabs, and *T. catappa* seeds [38, 103]. Their engagement with this behavior varies with the fluctuation in the tides and has only been documented in males. There is limited documentation of male Trinidad white‐fronted capuchins (*Cebus albifrons trinitaris*) using stones to open almond tree fruits (*T. catappa*) in an anthropically modified environment in the Ecuadorian Amazon as well [51].

### 4.3 Pan

Chimpanzees (*P. troglodytes*) are the most adept extant non‐human tool‐user, with a complex repertoire of tool‐using behaviors which are found to some extent in all studied wild communities [104, 105]. Their tool use is also considered to be culturally‐specific, as certain groups use tools in ways that are absent in other communities, even if the same tool substrate, or nutritional resource is available in both ecologies [50, 105]. For example, some groups in Bossou in Guinea, West Africa, and across the border into Liberia in Kpala, engage in pestle‐pounding, which entails using a young oil palm (*E. guineensis*) frond to pound the top of the palm tree and access the fibrous palm heart inside, a behavior which is not documented in other communities with oil palm trees [106].

The most common form of percussive lithic tool use in chimpanzees is a type of hammering aptly named nut‐cracking, wherein rocks (or in some communities, tree roots) are used to break open the shells of nuts so the kernel inside can be consumed [107]. Nut‐cracking involves a chimpanzee placing a nut on a stone or wooden anvil, which may or may not be mobile, then hitting it with a stone or wooden hammer [50, 107]. While chimpanzees live in numerous communities throughout western and central Africa, this behavior is only observed in some of the chimpanzee groups of West Africa, with the sites of Taï and Bossou being particularly well‐studied [105]. Similarly, even in communities of the same subspecies of chimpanzees (*P. troglodytes verus*) that all engage in nut‐cracking, there is still diversity in the types of nuts they crack and the tool material they chose for the task. Chimpanzees from Taï in Côte d'Ivoire, use immobile anvils in the form of tree roots or rock patches in combination with stone or wooden hammers, and vary their hammer type based upon the hardness of the nut they are attempting to crack, which include numerous species such as *Coula edulis*, *Parinari excelsa*, *Panda oleosa* and *Detarium senegalensis* [50, 108]. Conversely, the Bossou chimpanzees from Guinea exclusively use movable stone anvils and hammers, which are often transported short distances specifically to be used for nut‐cracking and they primarily crack oil palm (*E. guineensis*) nuts [107, 109].

The cultural diversity within chimpanzee subspecies and neighboring groups is particularly striking when comparing them to their sister species and only other extant member of the genus *Pan*, the bonobo (*P. paniscus)* [41]. While bonobos are capable of complex tool manufacture and use in captivity, they have infrequently been documented using tools, particularly in extractive foraging contexts, in the wild [110]. Their wild tool use primarily involves organic materials like sticks and leaves becoming part of communicative displays, covering themselves from rain, or in grooming/cleaning contexts, with some diversity in how habitual these behaviors are [111, 112]. The drastic difference in documented wild tool use between chimpanzees and bonobos may be due to fewer research teams studying them [104, 113]. It has also been suggested, however, that the rationale for tool use differs inherently between the two species. This distinction is thought to be the result of a disparity in the ecologies of the species, particularly in relation to nutritional resources, as bonobos tend to have fewer environmental barriers to nutritional success, resulting in their decreased intrinsic motivation for tool use [111]. On the whole, bonobos seem to have smaller tool repertoires than chimpanzees [110], a difference that is particularly interesting given that the two only diverged from each other around 1 Ma [113].

## 5 Defining Laterality

Many vertebrates express some form of behavioral lateralization, however, modern humans are an evolutionary singularity in the primate order due to the strong cross‐cultural bias for right‐handedness we display [2]. Up to 96% of humans use their right hand for skilled manipulation of objects [15]. Individual chimpanzees tend to have a manual specialization when engaging in complex tasks such as tool use, which appears similar at the individual‐level, but not identical to the handedness seen in modern human populations as they do not display the same population‐wide directional bias [114]. An individual chimpanzee may change which hand is preferred based on whether it is employing a tool, and what sort of tool is being used to accomplish the given task, something which is rare in humans [114]. It has been hypothesized that the switching of preferred hand during different types of tool use may stem from the varied cognitive and motor demands of the task, such that a chimpanzee prefers one hand for more delicate, dexterous movements like termite‐fishing, but prefers the other for more forceful behaviors like nut‐cracking [115]. In terms of percussive tool use, while young chimpanzees begin nut‐cracking ambidextrously, they show a 100% hand preference by the time they are ‘experts’ at the skill, something not demonstrated in other sorts of manual behaviors [107]. When engaging in percussive behaviors, macaques may use one or both hands depending on what they are attempting to open, whereas capuchins typically use both forelimbs to lift and throw hammerstones on the object [83]. Similarly, both capuchins and macaques often demonstrate individual manual specialization in standardized bimanual tasks [94, 116].

Comparisons between primate's percussive tool‐using behaviors have been proposed as a step towards, and compared to, the origins of knapping in the hominin clade [43, 82, 117, 118]. The relevance of laterality to this work stems from the fact that in humans, handedness is known to lead to asymmetric variation in the skeleton [15, 16]. Therefore, when individual chimpanzees and macaques use one hand more frequently for certain repetitive tasks such as percussive hammering, if their bones behave in a similar manner to humans, this potential manual specialization could cause small, yet quantifiable changes in osseous tissue of long bones of the preferred hammering hand compared to the less‐used hand. Conversely, in capuchins who engage in bimanual cracking, less directional asymmetries might be expected.

## 6 Relevance of Bone Morphology

Both cortical and trabecular bone can break while experiencing excessive loads, but are relatively resistant to routine stress and strain, helping to prevent breakage in normal use scenarios, as they can be fatal in the wild. The types and degree of strain varies between individuals due to their lifestyles, such that those experienced by athletes who throw with one arm at high speeds, or individuals who partake in certain types of manual labor, meaning their bone remodels to reflect the repetitive strains they personally experience [65]. The minimum effective strain (MES) constitutes the minimum amount of force needed to cause the mechanical force to be transmitted as a nervous signal [78]. Forces significantly surpassing the MES will cause microscopic damage to the osteon, the basic functional unit of bone, which then begins a modelling or remodeling process [78]. This process is referred to as bone's functional adaptation, which describes its ability to remodel in response to the mechanical loads it regularly experiences [23, 78]. Thus, recurrent biomechanical loading, such as during frequently lateralized behaviors like percussive tool use, could cause bone cells to preferentially resorb bone in areas that are not experiencing much strain, and deposit bone in areas enduring higher rates of strain [20, 23].

**Figure 4:**
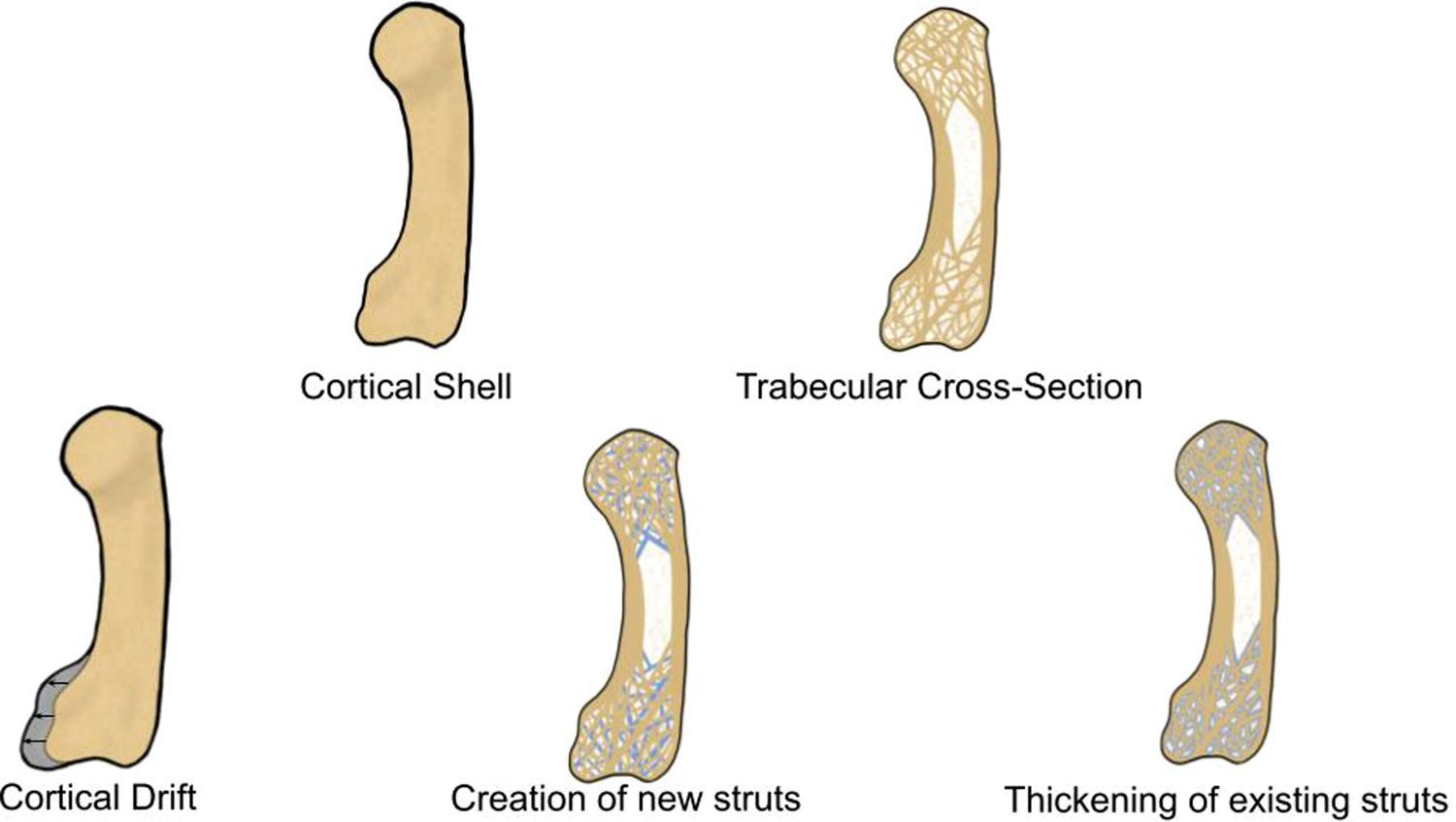
Bone Remodeling. Depicting the bone remodeling can change the internal and external structure of a bone, in this case, a metacarpal. For illustrative purposes only.

Over time, remodeling can cause changes to various aspects of both internal and external bone morphology, including cortical drift, thickening of existing trabecular struts, changes to the direction of struts, or creation new struts entirely, as seen in Figure 4, so the bone can better withstand the repeated type of loads it is enduring [9, 23, 78]. Infrequent tool use, and other sporadic actions which may result in significant, but inconsistent, forces are unlikely to cause significant changes to bone. By contrast, actions which repeatedly surpass the MES, such as pitching by elite baseball or cricket athletes, can cause significant changes to the macro‐ and microstructure of the bone, though there is some debate as to the differences in how trabecular bone may remodel between species and body regions [23, 28, 119].

Due to factors like cortical drift, the external features of long bones, such as enthesis size and shape, or the density of bone tissue can vary between species, as well as between individuals of the same species [9, 78]. Since bone reacts to these repeated mechanical loads in quantifiable ways, studying morphological variation may provide insights into the locomotor patterns and functional abilities, and potential tool‐use behaviors, that extinct hominins may have engaged in [77, 120]. This methodology of examining external bone morphology has been used for many decades, as it only requires evaluating the physical features of the bone's surface, rather than any additional analyses which may require cutting, drilling or otherwise damaging the specimen, while still providing numerous ways to examine bones of the upper limb of the same individual for asymmetries [29, 71, 121, 122]. For example, numerous studies have examined how various physical actions particularly associated with lateralized sports like tennis, cricket, or baseball can affect both the micro‐ and macrostructures of the long bones of the extremities asymmetrically [28, 119]. Furthermore, newer ways of quantifying variation in external morphology such as the validated entheses‐based reconstruction of activity (VERA) 1.0 and 2.0 methods, which algorithmically detect features on the entheseal surface, have been shown to be able to quantify the effects of experimental activity, including asymmetrically [26, 27, 69, 123]. While brief, these examples demonstrate that various aspects of bone morphology may reflect asymmetric changes, so rather than focusing on solely on one feature such as trabecular architecture or entheseal morphology, understanding how the whole bone may display the effects of behaviors like percussive tool use is critical to determining which method may be most applicable to the fossil record. It should be noted however, that when examining fossil specimens for asymmetries, recognizing potential impacts of taphonomic distortion because of fossilization, pathological or age related changes, and burial is crucial [124].

## 7 Proposed Case Study: Nut‐Cracking in Chimpanzees

As discussed in previous sections, multiple wild primate species engage in the use of lithic tool complexes, however, most long‐term studies of this phenomenon have occurred in nut‐cracking chimpanzees. For this reason, they serve as a valuable starting point for research into the effect of manual specialization and potential resultant asymmetries in primate bone. Chimpanzees have a complex understanding of the mechanical properties of the tools they utilize and there is individual variation in the efficiency with which they crack these nuts [7, 83, 84]. Similar evidence of tool selection and material property knowledge has been demonstrated in capuchins [125]. Nut‐cracking is one of the most complex tool‐use practices observed in non‐human animals, and as a result, it takes many years for young chimpanzees to master, although there is some debate as to whether the mechanism of learning is via observational social learning, imitation, or individual learning and practice [50, 84, 85, 126].

Nut‐cracking is estimated to have been transmitted across 200 generations of chimpanzees, as lithic assemblages have been discovered in the home of the Taï chimpanzees, Côte d'Ivoire, dating back 4300 years, and both lithic and organic remnants of these behaviors can be found in the archaeological record [127, 128]. For the Taï chimpanzees, tool use is thought to be a daily occurrence [108]. The Taï chimpanzees start learning to crack nuts around 3–4 years old and primarily crack nuts during the 4‐month “coula season,” which coincides with the dry season, making the nuts an important component of the chimpanzee's diet [108]. The other most‐studied nut‐crackers are the chimpanzees of Bossou, located in Guinea [107]. The Bossou chimpanzees begin learning to crack nuts between the ages of 3–4 years old with a critical period from 3 to 7 years, which, if missed, means the chimpanzee is unlikely to ever acquire the skill [104] but see [59]. Both the anvils and hammers are movable stones, typically made of granite, quartz, or diorite, and are often transported short distances specifically to be used for nut‐cracking [107, 109]. Nut‐cracking occurs year‐round in Bossou, with oil palm (*E. guineensis*) being the most frequently cracked nut [107].

Due the differences in the ways the Tai and Bossou nut‐cracking communities employ and choose their tools, along with evidence that the behavior cannot be individually reinvented, nut‐cracking is considered to be culturally specific to the groups that practice it and as such, is passed on via a type of cultural transmission [129]. Importantly, because of modern chimpanzees' close evolutionary relationship with humans, their use of tools, and their culturally varied implementation of percussive instruments, they can be an incredibly useful proxy for the study of the evolution of percussive tool use in potential human ancestors [12, 104]. By comparing the ways humans and chimpanzees create and interact with tools, we can learn about which traits might be plesiomorphic (i.e., ancestral and inherited from the last common ancestor (LCA) of the two species). Additionally, by connecting actions with resulting morphological traits, it can help us infer behavior when similar morphology is described in extinct species.

Nut‐cracking has also been suggested as a potential precursor to early stone‐flaking in the hominin lineage [8, 42, 81, 117, 118, 128]. The action requires a significant amount of power to crack the shell, but not so much that the kernel inside is shattered, which creates meaningful forces on the hand holding the hammerstone [118]. This could potentially cause enough mechanical strain to active bone's dynamic remodeling, thus causing morphological changes in the bones of nut‐crackers. Furthermore, as chimpanzees are considered fully lateralized once they have mastered nut‐cracking, as they will only use one hand to hold the hammerstone, there could be a quantifiable directional asymmetry, or an osteological signature caused by nut‐cracking behaviors, particularly in the communities that engage in nut‐cracking year round [72, 84, 107]. Quantifying what the potential ‘damage signature’ left by the repetitive, forceful act of nut‐cracking has on the hand bones of chimpanzees has not yet been done. While some research has investigated and compared the microarchitecture of the metacarpals and carpals in bonobos, chimpanzees, orangutans, gorillas, and modern *H. sapiens*, these studies have often focused on a single bone, on a single side, and they have not analyzed these specimens in the context of tool‐using behaviors [64, 130]. Thus, examining bones of both hands of nut‐cracking primates and comparing them to non‐nut‐crackers can help answer the question of when do repeated, forceful actions, such as nut‐cracking, cause quantifiable, morphological change to bone in non‐human primates? [131].

Importantly, this sort of research in inherently comparative in nature; to determine whether one hand is impacted by percussive tool‐use behaviors, both hands of the same individual must be studied. However, scanning both sides of the same individual is not current practice in the field. A key example of this issue is present in a new dataset meant for comparative morphology in hominoid and anthropoid primates, which collected a massive amount of data from over 386 individuals from 47 genera [132]. While this is an incredible collection, it lacks bilateral data from of most individuals, essentially limiting these scans from being used in any asymmetry studies until, or unless, the other sides of those individuals are scanned. Current scanning and analytical practices essentially ignore any potential for directional or fluctuating asymmetries in the primate order, inherently assuming a lack of asymmetry. However, limited previous work has shown that while humans are more strongly lateralized both in terms of strength and direction than any other primates, directional asymmetry can be quantified in bones of the upper limbs of other species, such as chimpanzees, so it can likely be identified in species who have not yet been examined in this manner [19, 71, 72]. Furthermore, a recent experimental study of rats who were exposed to asymmetric loading demonstrated that this induced asymmetrical changes which could be detected via numerous methods of analysis, including in cross‐sectional geometry, increased cortical thickness, changes to external morphology such as diaphyseal shape and entheseal areas, as well as improved biomechanical function in the stimulated limb [69]. This serves as proof of concept that limb preferences which create asymmetric variation can be quantifiable via the proposed methods even in incredibly distantly related species [69].

Numerous studies have analyzed each of the metacarpals when attempting to determine extinct hominin's manual capabilities, so examining the effect of percussive tool‐use on the metacarpals in primates would be in line with current morphological practice [63, 64, 67] especially as directional asymmetries have previously been identified in these bones [19]. Particular attention should be given to the pollical metacarpal as it plays a critical role in primates' grasping abilities and is a focus of research into potential tool‐use abilities in extinct hominins [20, 64, 68]. Prior studies have rarely identified handedness‐related directional asymmetries in the carpals' trabecular and cortical structure in humans, so these bones likely do not need to be the focus of this research [78, 80]. To be used for quantification of variation potentially resultant from percussive tool use in both the internal trabecular and external cortical morphology, high‐resolution microcomputed tomography (microCT) scans from both hands of adult chimpanzees should be acquired, as changes may be more apparent in the former due to its faster turnover rate, and the ability to measure the degree of and direction of anisotropy. Other scanning methodologies, like photogrammetry or structured light scanners can be used to quantify external, cortical asymmetries, such as entheseal shape and size, which can be affected by differential muscle use and are thus beneficial for understanding differential aspects of these behaviors on bone [72].

**Figure 5:**
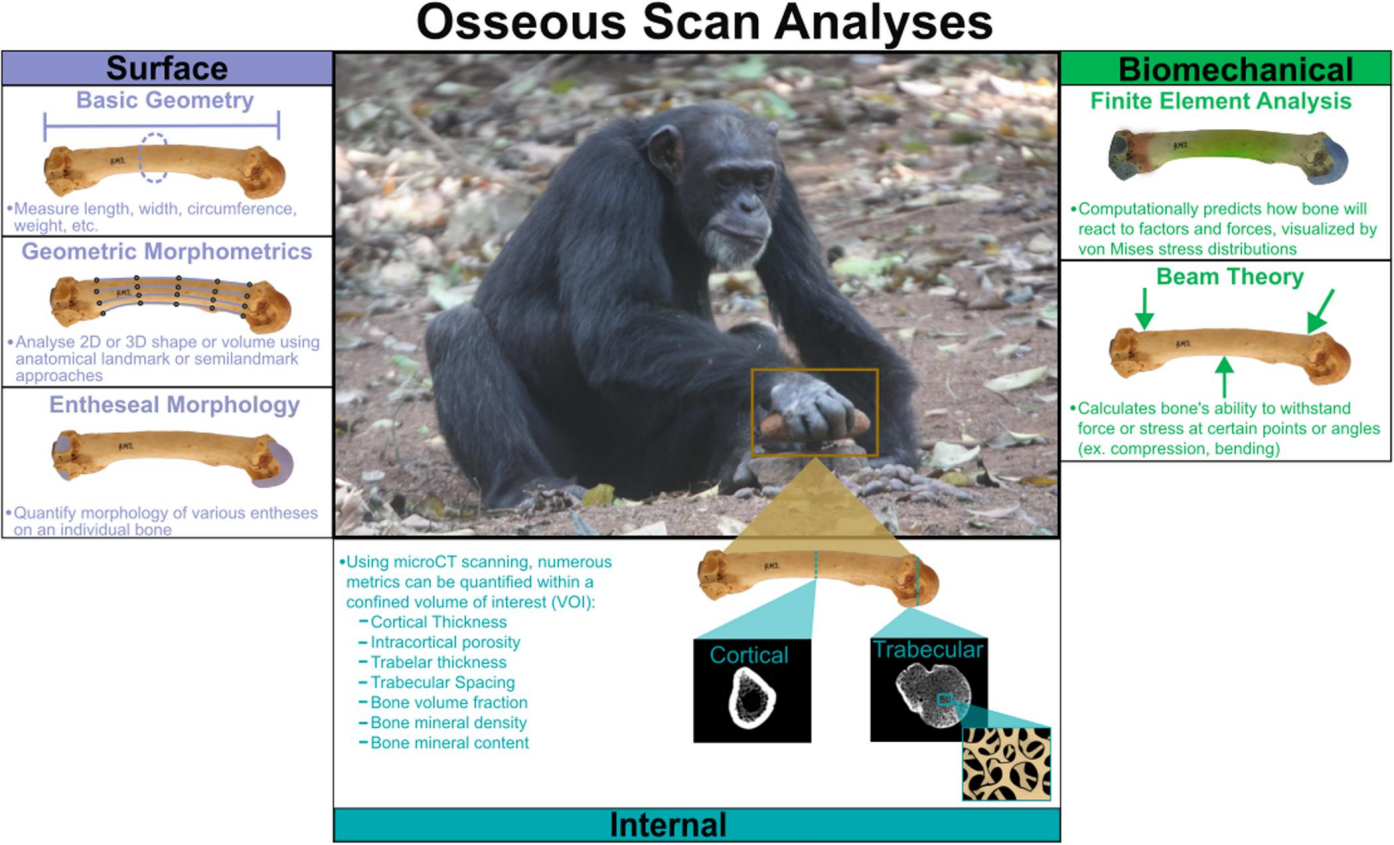
Summary of potential osteological analyses to analyse asymmetries related to percussive tool‐use. All images for illustrative purposes only, and do not depict actual analyses. Chimpanzee photo credit to T. Matsuzawa/Kyoto University Primate Research Institute. Metacarpal photo taken by K. Almeida‐Warren. For further methods on basic geometry refer to: Sarringhaus et al. [70]. For further information on geometric morphometrics: Morley J et al. [133] For further methods on entheseal morphology: Karakostis and Hotz [65]. For further methods on Finite Element Analysis: Bucchi et al. [67]. For further methods on beam theory: van Lenthe GH et al. [134] For further methods on examining internal morphology: Dunmore CJ et al. [135] For further methods on the VERA 2.0 protocol: Karakostis [123].

Scans of chimpanzees from communities like Taï and Bossou will be a critical dataset for this research, as they have well‐documented life history information such as preferred hand during various forms of tool use, are prolific nut‐crackers in the wild, and for whom skeletal collections exist [131]. Results could then be compared to the scans of individuals who come from similarly long‐studied communities, but who do not engage in nut‐cracking like Budongo, or Ngogo. An alternative comparative dataset could come from museum collections or captive environments like zoos and research centers. However, the former would likely have less life history data on individuals and as such, their nut‐cracking history would be an unknown and thus uncontrolled variable. The results from the known nut‐cracking chimpanzees can be used to create a scale nut‐cracking “damage,” which scans of unknown history can be compared to. As demonstrated in Figure 5, numerous types of analyses can be used to investigate potential asymmetries in the bones of nut‐crackers. Importantly, rather than focusing on only one feature of bone, or one type of analysis, different methodologies including trabecular analyses, geometric morphometrics and entheseal shape should be applied, as they have previously effectively detected relevant asymmetries [19, 28, 69, 73] and may reveal different degrees and types of asymmetries.

Chimpanzees, like humans, are typically somatically mature by the time they begin reproducing, which is often in their early‐ to mid‐teens [113]. In female chimpanzees, this corresponds with the time they disperse from their natal group [113]. Immature bone is more disorganized than adult bone and has not yet experienced enough remodeling for trabecular struts and haversian canals to be aligned in response to the typical forces [23]. Thus, skeletally immature individuals should likely be excluded from these analyses. Similarly, older chimpanzees may begin to show degeneration of their bone, or osteopenia (decreased bone density) as bone tissue is resorbed faster than it is deposited [136]. In females, the risk of this increases during menopause, a life history milestone which has recently been demonstrated in chimpanzees [137]. However, these individuals should not be excluded unless the bone mineral density and bone mineral content measures are outside the standard deviation of the other scans.

A potential limitation to this sort of research stems from the fact that humans are the only obligately bipedal primate, meaning our forelimbs are not subjected to regular locomotor loads. It has been suggested that as a result of the locomotor decoupling of our limbs [138], directional asymmetry in human forelimbs may be easier to recognize and quantify than in quadrupedal species [121, 139]. While all great apes are considered to have orthograde body posture, chimpanzees, bonobos, and gorillas are quadrupedal knuckle‐walkers, whereas orangutans, although rarely terrestrial, are typically palmigrade. Similarly, anthropoids such as capuchins and macaques are pronograde quadrupeds. Previous research has demonstrated various aspects of skeletal morphology in both the forelimbs and hindlimbs of primates does not solely reflect locomotor loading patterns [79], and are also affected by factors such as genetics and manipulative repertoires [66]. This means that while osteological changes because of percussive tool use may be more obvious in our own highly‐lateralized, bipedal species, it does not negate that similar signals may be detectable to a lesser degree in other primates. Thus, seeing whether and how osseous asymmetries vary between these species with differing locomotor patterns can help us understand the interplay between various factors that can lead to bone remodelling, including, but not limited to locomotion, manual behaviors, body size, and phylogeny.

Furthermore, our intention is not to attribute any potential asymmetries found to tool use in isolation, but rather to propose that it is possible to explicitly model asymmetry as a function of tool use while simultaneously accounting for other covariates known to influence skeletal morphology (i.e. locomotor behavior, phylogeny, body size, age, etc.). By adopting a hierarchical modelling framework, these factors can be incorporated as either fixed and random effects, allowing the unique contribution of percussive tool use to be estimated while partitioning variance attributable to other biological and evolutionary processes. This comparative approach is specifically designed to disentangle the influence of tool use from potential confounding factors, rather than assuming that asymmetry is solely driven by behavioral loading.

While chimpanzees are a strong candidate for initial research into the effects of percussive tool use on primate bone, the relevance of this research can only be improved by applying it to multiple different primate species. By extending this research to include other percussive tool using species like macaques and capuchins, as well as extant hominids not known to engage in percussive tool use, a broader understanding of how manual abilities affect, or are reflective of directional asymmetries can be elucidated. Similarly, by incorporating data from species with different behavioral repertoires (i.e. orthograde v. pronograde posture, primarily arboreal v. terrestrial ecology, extent to which they engage in other extractive foraging or tool‐use behaviors) will provide additional statistical support for whether directional asymmetries are likely to be the result of percussive tool use or is more likely the result of other aspects of the primates' activity patterns. Importantly, by quantifying whether and what type of asymmetry is present in various primate species, predictive models which integrate these various ecological and behavioral factors can be created, which can then be applied to fossil remains. Thus, by understanding the degree of bilateral asymmetry in the hand bones of our closest living relatives, and particularly whether it occurs to a higher degree in chimpanzees who engage in a percussive tool use behavior like nut‐cracking, could add another method for examining tool use behavior in the archaeological record.

## 8 Future Directions and Applicability to the Fossil Record Via Machine Learning

Conceptually, there are numerous future directions this work could go in, but future studies should adopt a multi‐proxy approach to distinguish changes in bone morphology arising from lifetime mechanical loading from those reflecting inherited evolutionary adaptations. Integrating behavioral observations with experimental validation, as well as virtual anthropology methods [140] such as finite element analysis, geometric morphometrics, cortical and trabecular bone quantification, and other complementary imaging and biomechanical techniques could provide a more comprehensive understanding of how habitual loading influences skeletal morphology. These approaches should be applied at both the intra‐ and interspecific levels to separate within‐lifetime plastic responses from evolutionary patterns across taxa. Another promising direction would be to simulate bone apposition and resorption in silico under different loading regimes and compare the predicted remodeling patterns with those observed in vivo [141]. Electromyography is another relevant method, which has been used in recent work to examine aspects of muscle recruitment in humans when making and using stone tools [142, 143]. This can provide insight into which muscles are used most when engaging in percussion and pinpoint which entheses are most likely to be affected by these activities in non‐human primates [142, 143]. Similarly, natural experiments, such as comparisons between percussive tool‐using and non‐tool‐using populations of the same species (e.g., nut‐cracking vs. non‐nut‐cracking chimpanzees or macaques), or between captive and wild individuals with contrasting mechanical loading histories, could help disentangle behavioral plasticity from evolved morphological traits by applying phylogenetic comparative tools to these datasets. Together, these complementary approaches would provide a stronger framework for interpreting skeletal signatures of tool use in extant primates and, ultimately, the fossil hominin record.

Critically, once the morphological impact of percussive tool‐use has been quantified on bone in numerous individuals of different primate species, including those who were and were not known percussive tool‐users, powerful approaches such as machine learning (ML) classification techniques can be used to categorize other specimens, such as hominin fossils, as being tool‐users v. non‐tool‐users. This could be done in a variety of ways, as ML approaches can be applied to multiple tool use proxies extracted from geometric morphometric data, such as shape or size asymmetries. Using the results of how percussive tool use affects primate bone in different clades to train machine learning models could likely increase the accuracy with which the models can predict whether various fossil hominins show potential osteological evidence of tool use. Alternatively, the results could help train models to predict the extent of microdamage caused by percussive hammering that must be present in different species to be detected in the fossil record. Essentially, these data could be used as predictors in either ML regression or classification tasks and thus can help improve our understanding of percussive tool use in fossil species. This sort of ML based classification methodology has been used previously to identify damaged versus undamaged areas on wooden tools caused by wild chimpanzees during nut‐cracking from 3D models with 96.3% +/−1.4% accuracy [12]. This study then used models to create “heat maps” of surface damage, which helped to delineate between regions of wear on the tools [12].

Similar methodology has previously been used to help discriminate the most likely locomotor repertoire of fossil primates [144]. By training models with both the tool‐holding and non‐tool holding hand of known percussive tool‐users, as well as non‐percussive tool‐users of various species, there is a lower likelihood of overfitting [12, 145]. Numerous ML classification algorithms should be employed and then the performance of classification of tool user type can be compared for accuracy via cross‐validation [145]. Furthermore, if behavioral data is integrated with the osseous effects of tool use in the various primate species known to use percussive tools (e.g., capuchins, macaques, chimpanzees), phylogenetic comparative methods can be used to extrapolate the likelihood of percussive tool use in extinct hominins, and more broadly, hominid taxa, while accounting for the phylogenetic non‐independence of these species [146]. Thus, having examples from varied primate species and infraorders can allow phylogeny to be accounted for in these models. Examinations into these features in the hands of both modern and ancient *H. sapiens*, as well as other hominins, may also allow us to determine when the cross‐cultural right handedness bias evolved as well [2, 15].

## 9 Conclusions

Researchers have extensively studied the behavior of percussive tool‐use in several primate species and have investigated the physical effects of these behaviors on the tools used [12, 81, 128]. However, studying the hand bones of percussive tool‐users themselves to determine whether lasting changes to bone morphology occur as a result of this behavior would be novel [131]. Analyzing the bones of both hands of individual primates who engaged in percussive tool use, as well as individuals who do not engage in this behavior can provide critical insight into the interplay between manual behaviors and hand morphology. Furthermore, this sort of investigation could fill a void in the current literature as the first to examine asymmetries in the hammering versus non‐hammering hand for the effects of percussive tool‐use in primates. Both the bones of non‐percussive tool using primates, and the non‐hammering hands of percussive tool‐users can serve as controls in this sort of study. Creating a better understanding of the effects that percussive tool use has on the hands of primates, who may use tools in ways similar to extinct hominins, means this research provides insight into the degree and range of percussive tool‐use evolution in the hominin clade.

By comparing the internal and external morphology from both upper limbs of the same chimpanzees, this sort of study could help determine whether the degree of lateralized behaviors demonstrated by individual chimpanzees causes asymmetrical variation at the skeletal level. If the expected long‐term remodeling from behaviors like‐nut cracking is found asymmetrically, this could affirm current evidence that wild chimpanzees show individual‐level manual specializations, and population‐level directional asymmetries could support broader concepts of handedness in non‐human species [114, 115, 116]. Even if no significant changes are found in the preferred hand versus the non‐preferred hand of percussive tool users, this will still be an important result. This could mean that even though highly lateralized behaviors can induce morphological change in human bone, it may not in other primates. This could be because the MES in primates is higher than in human bone, meaning that percussive hammering may not affect their morphology in the same way it does in humans and as a result, signatures of remodelling may not be apparent at all. Alternatively, the level of MES in humans could be a derived trait, which resulted from our bipedality. Because we do not bear weight on our hands like knuckle‐walking chimpanzees while ambulating, the routine loads our hands endure are likely lower than that of chimpanzees and other primates. Knowing this would also be important for extrapolation to the paleoanthropological record, as it could mean that other obligate bipeds may show more evidence of tool use than more occasional bipeds in the bones of their hands even if they engaged in the same amount of percussive tool‐use. Ultimately, if it is possible to provide a better understanding of the effect that tool use has on the hands of primates using this methodology, this research can potentially provide insight into the degree and range of percussive tool‐use in human clade.

## Supporting information


Supporting File

## Data Availability

Data sharing not applicable to this article as no datasets were generated or analysed during the current study.
