# Supplementary material for: Impacted Bones: Can Extant Primates Help Identify Tool Use in Early Hominins?
Source: Evol Anthropol. 2026 Jul 19;35(3):e70042. doi: 10.1002/evan.70042 (PMC13382194; doi:10.1002/evan.70042)
Supplement: Supplementary file 1 — Supporting File [file EVAN-35-e70042-s001.docx]

**Glossary**

*Directional asymmetry –* the systematic and regular variance between two halves of a bilaterally symmetric organism, wherein one side develops larger or differently than the other in a predictable fashion, such as the additional lobe of the right lung

*Entheses –* the sites on bones where connective tissues such as tendons or ligaments attach

*Handedness –* a population-level directional bias in terms of manual behavior wherein individuals are specialized to use one hand or the other (e.g. *Homo sapiens* as a species are right-handed, but individuals may be left-handed)

*Laterality –* a broad concept which includes any asymmetry in the use or function of bilaterally symmetrical structures (e.g., brain hemispheres, hands, feet, eyes)

*Manual specialization –* consistent use of one hand by an individual, particularly for complex or demanding tasks. However, individuals may specialize with different hands, meaning a population-level bias may not be detectable.
